# Tropospheric Ozone Concentration on the Territory of Russia in 2021

**DOI:** 10.1134/S1024856022060033

**Published:** 2022-12-15

**Authors:** V. V. Andreev, M. Yu. Arshinov, B. D. Belan, S. B. Belan, D. K. Davydov, V. I. Demin, N. V. Dudorova, N. F. Elansky, G. S. Zhamsueva, A. S. Zayakhanov, G. A. Ivlev, A. V. Kozlov, L. V. Konovaltseva, S. N. Kotel’nikov, I. N. Kuznetsova, V. A. Lapchenko, E. A. Lezina, V. A. Obolkin, O. V. Postylyakov, V. L. Potemkin, D. E. Savkin, I. A. Senik, E. V. Stepanov, G. N. Tolmachev, A. V. Fofonov, T. V. Khodzher, I. V. Chelibanov, V. P. Chelibanov, V. V. Shirotov, K. A. Shukurov

**Affiliations:** 1 Peoples’ Friendship University of Russia, 117198 Moscow, Russia; 2 V. E. Zuev Institute of Atmospheric Optics, Siberian Branch, Russian Academy of Sciences, 634055 Tomsk, Russia; 3 Polar Geophysical Institute, Russian Academy of Sciences, 184209 Apatity, Russia; 4 Obukhov Institute of Atmospheric Physics, Russian Academy of Sciences, 119017 Moscow, Russia; 5 Institute of Physical Material Science, Siberian Branch, Russian Academy of Sciences, 670047 Ulan-Ude, Russia; 6 Prokhorov General Physics Institute, Russian Academy of Sciences, 111933 Moscow, Russia; 7 Hydrometeorological Center of Russia, 123242 Moscow, Russia; 8 Vyazemsky Karadag Scientific Station – Nature Reserve of Russian Academy of Sciences – Branch of Kovalevsky Institute of Biology of Southern Seas of Russian Academy of Sciences, 298188 Feodosia, Russia; 9 State Nature Organization Mosecomonitoring, 119019 Moscow, Russia; 10 Limnological Institute, Siberian Branch, Russian Academy of Sciences, 664033 Irkutsk, Russia; 11 Instrument-Making Enterprise OPTEC, 199178 St. Petersburg, Russia; 12 Typhoon Scientific and Production Association, 249038 Obninsk, Kaluga oblast Russia

**Keywords:** atmosphere, air, concentration, ozone, maximum permissible concentration, surface layer, troposphere

## Abstract

Ozone is one of the most toxic admixtures in the troposphere. Therefore, it is among the main pollutants and its concentration is monitored. This work represents an overview of continuous measurements of the ozone content in the troposphere on the territory of Russia throughout 2021 carried out on an initiative of scientific and educational institutions at 17 stations in different Russian regions. The monitoring results showed that the daily average ozone concentration exceeded the MPC_d.a_ level during a major part of the year at all observation sites, and by a factor of two or even three at a number of stations. At six stations, concentrations in excess of the maximum permissible one-time concentration MPC_m.o_ were recorded. This requires a more comprehensive analysis of the composition and concentration of ozone precurcors and the development of measures to reduce their emission into the atmosphere.

## INTRODUCTION

The urgency of studying the tropospheric ozone stems from its physicochemical properties [1–5] and its effect on biological entities and environmental structures after its content increases in air.

As biological and medical studies have shown, ozone in the troposphere is a virulent poison exhibiting, in addition to the general toxic effect, such properties as mutagenic and carcinogenic potentialities and radiomimetic effect (an action on blood similar to ionizing radiation) [2–4]. Based on [6], 30-min inhalation of ozone at the concentration 0.8 mg/L is equivalent to a 100-R dose. Ozone is even more toxic than such a well-known poison as hydrocyanic acid. Therefore, it is classified as a class one hazardous substance in regulatory documentation [7].

In high concentrations, ozone strongly inhibits the activity of plant life. Plant response to the increased ozone concentration is reduced productivity and even death in some cases. Calculations of American scientists [8] have shown that the economic losses from reduced crop productivity are from 1.9 to 3.3 trillion dollars yearly in the United States. Analogous losses for a part of southeastern Asia are 68 billion dollars [9]. In addition to crop productivity reduction, ozone also decreases the uptake of carbon dioxide by vegetation, which can lead to enhancement of the Earth’s radiative forcing [10, 11].

In addition to the effects mentioned above, ozone is the strongest oxidant capable of destroying rubber and caoutchouc and oxidizing many metals, even from the platinum group [12–16].

With its long (from few days to few months) lifetime in the atmosphere and strong solar radiation absorption, tropospheric ozone plays an important role in the greenhouse effect. Estimates [17] indicate that it contributes more than 8% of the total air heating due to absorption of solar radiation by greenhouse gases. Later estimates show that this contribution may be even larger.

This variety of possible adverse consequences from an increasing concentration of tropospheric ozone for both human beings and the environment call for closer attention to the trends in variations in its content in surface air. This gas is considered the number one air pollutant in all developed countries. The authors of work [18] mentioned that there were over 10 thousand stations for monitoring ozone and its precursors in Europe as early as 2003. Most important is that the information is made available to the population and used in decision making by governing bodies. The United States and Europe have already succeeded in reducing ozone concentrations in air. For instance, based on data from 119 stations in Great Britain, the measures taken resulted in a reduction of the concentration of surface ozone from 1980 to 2019 by a factor of 2–6 depending on the region [19]. The United States managed to reduce the emissions of ozone precursors by a factor of two [20, 21]. China undertook similar efforts; however, a significant reduction of the emissions of ozone precursors could be achieved only in certain industries [22–24].

The former Soviet Union and present-day Russia did not pay due attention to monitoring and measures for reducing the ozone content. Rosgydromet, entrusted with the responsibility for monitoring ozone content, is proceeding with the technological modernization of the observational network, and so far is measuring surface ozone in just a few large and industrial cities. The two biggest megalopolises in Russia, St. Petersburg and Moscow, have competitive systems for monitoring surface ozone and other pollutants. An ecological monitoring network of State Nature Organization Mosecomonitoring has been operated in Moscow since 2002, which is a specially authorized governmental ecological monitoring organization in Moscow [25]. The surface ozone concentration is monitored at 17 automatic air pollution control stations (AAPCS) hourly and around-the-clock. The 20‑min averages are stored in a database. The Mosecomonitoring network stations carry out measurements using gas analyzers of three types based on ultraviolet photometry: Casella Monitor ME 9810B, Environnement S.A. O3 42M, and HORIBA Ltd. APXA-370 model APOA-370, and a OPSIS AB AR500 analyzer, based on differential optical absorption spectroscopy (DOAS). The instruments are included in the State Register of Measuring Instruments and certified by the State Meteorological Service. Analytical materials on the state of the environment in Moscow are annually reported [26]. However, data on the content of surface ozone on the territory of Russia are still not provided in state reports [27, 28]. In the rest of Russia, the ozone observations are carried out on an initiative basis, mainly by scientific or higher-education institutions.

The purpose of this review is to inform the scientific community about the ozone content in the surface air layer in 2021, and about the causes for its variations and the compliance of ozone concentrations recorded at different monitoring sites to national hygienic standards [7].

In this review, we used the data obtained by the coauthors at 17 sites in Russia, differing by their geographic and climatic characteristics, as well as by the anthropogenic load on the environment. The spatiotemporal variations in the surface ozone on the territory of Moscow are analyzed using averaged measurements at Mosecomonitoring AAPCS of two types: seven urban AAPCSs and four traffic AAPCSs (https://mosecom.mos.ru/vozdux/); it is noteworthy that the maximal ozone concentrations are represented by highest hourly average concentrations recorded at all AAPCSs.

It should be noted that, like previous half-year reviews in 2020 [29, 30], 2021 coincided with the period of coronavirus pandemic and, as such, can reflect the lockdown results. The meta-analysis carried out in [31–33] using monitoring at tens of stations around the globe showed that reduction of emissions of the main admixtures was usually accompanied by the growth of ozone concentration in the surface air layer. Interestingly, ozone concentrations decreased in the free troposphere during the pandemic [34, 35]. There was no aim of elucidating the lockdown consequences in the review, because this requires the data for previous years unavailable at a number of stations. Here, the changes in the ozone concentrations in the free troposphere are verified using results from aircraft sensing.

## 1 NEW STATIONS AND INSTRUMENTS USED

The total set of stations and the instrumentation installed at these stations, as well as the operational modes and calibrations, were listed in previous reviews [29, 30]. In 2021, we resumed measurements at stations Slyudyanka and Tarusa, opened new OPTEC stations in Karelia and Boyarsky settlement in Buryatia. In this section, we describe these stations.

The atmospheric monitoring station Listvyanka (51°50′48″ N, 104°53′58″ E, 670 m ASL) is located in Irkutsk oblast on the southwestern coast of Lake Baikal, in a region of the source of the Angara River, at the top of a coastal hill (200 m above the lake level) on the territory of the Astrophysical Observatory of the Institute of Solar-Terrestrial Physics, Siberian Branch, Russian Academy of Sciences. The nearest settlement Listvyanka is 2 km off the station on the coast of the lake. The location of the station at the top of the hill makes it possible to escape the effect of local sources of atmospheric pollution (settlement, motor vehicles) and to track the regional transports of pollutants, primarily from the direction of Irkutsk, Angarsk, and Shelekhov. The complex of automatic gas analyzers year-round monitors the presence in the atmosphere of different admixtures, including ozone, a new optical ozone analyzer F-105 (OPTEC, St. Petersburg, Russia) was installed at the station in February 2021. Since 2001, the station had become a participant of the International Program “Acid Deposition Monitoring Network in East Asia” (EANET); and the data from the station are planned to be made available on the Internet.

Under the auspices of the Peoples’ Friendship University of Russia (RUDN) and the Prokhorov General Physics Institute, Russian Academy of Sciences (GPI RAS), Russian Academy of Sciences, an automatic station for monitoring surface ozone and the main meteorological parameters started operating in summer 2021 in Tarusa, Kaluga oblast. The station is located on the territory of Tarusa branch of GPI RAS (54°43′36″ N, 37°10′40″ E, 128 m ASL) situated at the center of the city in the residential building zone 350 m away from the coast of the Oka River. Tarusa is 110 km south of Moscow on the high bank of Oka bend, surrounded by pine forests in the north. Its population is slightly more than nine thousand residents. Tarusa and its surroundings have no industrial plants and, as such, are considered as one of the resort regions in the far Moscow oblast. The main local sources of anthropogenic pollution of the atmosphere are motor vehicles and municipal services. The nearest busy motorway Serpukhov–Kaluga is 1.5 km away from the center of Tarusa. The distances to the nearest bigger cities are: ∼30 km to Serpukhov, ∼70 km to Kaluga, and ∼80 km to Tula. The monitoring station is equipped with a chemiluminescent ozone analyzer 3.02P-A (OPTEC) with a sensitivity of ∼1 μg/m^3^. Sampling is carried out via Teflon pipes at an altitude of 5 m above the Earth’s surface. The measurements are performed in the continuous long-term monitoring mode. Current values of the parameters measured are recorded once a minute, followed by their averaging over 20 min and storing the result in the database.

The atmospheric monitoring station OPTEC-Karelia is in the settlement Voloma (63°44′41″ N, 31°56′33″ E, 185 m ASL) in the northern part of the Republic of Karelia, about 80 km away from the border with Finland (Russian Far North). The settlement is in a depression surrounded by hills up to 100 m in height. Outside the settlement there are large forest massifs as well as hundreds of small lakes. The average air temperature is −28°С in winter and +25°С in summer. The lowest observed temperature reaches −50°С in winter and the highest temperature is +40°С in summer. The wind direction at the location of the station is predominantly S–W and the wind speed is 2–3 m/s. The annually average pressure does not exceed 738–740 mmHg. The average height of snow cover is 1.2–1.5 m. There are no big industrial plants in the region; the nearest, Segezha Pulp and Paper Mill (PPM) is 110 km away, and the Kostomuksha ore mining and processing enterprise is 114 km away. Quite rarely, a bad smell from cellulose processing at the Segezha PPM is detected when the wind is strong. There is small industrial plant for wood production and drying in the settlement. Temperature inversions in the atmosphere are observed mainly in winter owing to the specific orography at the location of the settlement and predominant type of anticyclonic weather. This leads to intense accumulation of pollutants (СО and CO_2_) in the surface air layer. For instance, the average concentrations are 200 μg/m^3^ for CO and 400 mg/m^3^ for CO_2_ during summer, and 800 μg/m^3^ for СО and 1200 mg/m^3^ for CO_2_ during winter. The accumulation of the carbon oxides in the surface air layer is likely due to the specific properties of the fuel used in the settlement: wood-burning stoves are used in the local boiler and in private houses. The OPTEC-Karelia station has operated since May 2021 in the pilot mode; it comprises the channels of measuring the concentrations of О_3_ (ozone), СО (carbon monoxide), CO_2_ (carbon dioxide), and ^1^Δ*g*(O_2_) singlet oxygen. The surface concentrations of ozone and singlet oxygen are measured using domestic solid-state chemiluminescent analyzers mod. 3.02P-A and 102-А, respectively. The limiting values of the main error of the 3.02P-A analyzer measurements are ±20% for the range of 0–30 μg/m^3^ and ±20% for the range of 30–50 μg/m^3^. The limiting values of the main error of the 102-A analyzer measurements are ±20% for the range 0–10 μg/m^3^ and ±20% for the range 0–200 μg/m^3^.

Boyarsky station is located on the southeastern coast of Lake Baikal, 160 km away from Ulan-Ude. This region is characterized by large temperature contrasts between the lake and adjoining territory, intensifying due to the closed position of Lake Baikal, surrounded in all directions by mountain ridges. The temperature gradient between the lake depression and adjoining dry hollows, reaching 20°С and more, is one of the main factors of formation and development of intrahollow circulation and its propagation into the lake basin, often favoring accumulation of atmospheric pollutants. Boyarsk village can be considered to experience weak anthropogenic impact: certain effects can be due to small industrial centers (Babushkin (22 km), Kamensk settlement (50 km), Selenginsk settlement (60 km), and others). A mixed forest (birch, pine, and cedar) lies in the immediate vicinity of the station. The concentration of surface ozone was measured using a 3.02 P-A chemiluminescent gas analyzer. The instrument was calibrated using a Mod. 8500 Monitor Labs calibrator. The observations at Boyarsky station were episodic, in the period of expeditionary works (April 13–18 and July 21–August 20, 2021).

## 2 MEASUREMENT RESULTS

### 2.1 Annual Average Data

**Fig. 1.**
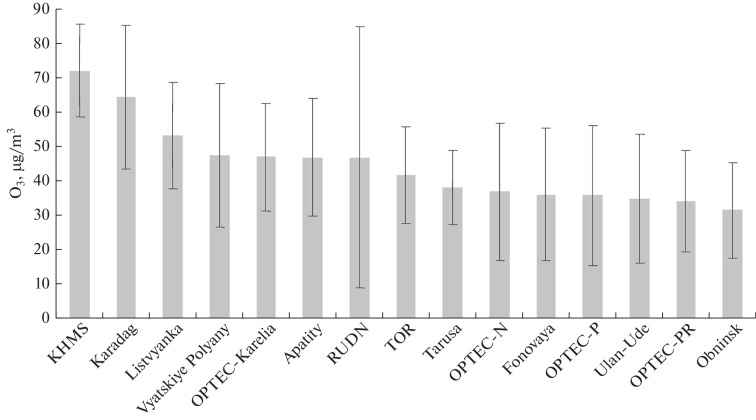
Annual average ozone concentrations at Russian stations.

Data on annual average ozone concentrations in the surface air layer at all stations that conducted measurements in 2021 are presented in Fig. 1.

On the one hand, Fig. 1 shows that the annual average ozone concentration is higher than 30 μg/m^3^ at all stations. This is even larger than the daily average maximum permissible concentration (MPC_d.a_), indicating that the norm is exceeded throughout the year [7].

On the other hand, with respect to the annual average concentration, the stations line up in quite an intricate order. The concentrations are the largest at Kislovodsk high-mountain station (KHMS), in Karadag, and in Listvyanka, i.e., at locations far removed from anthropogenic sources of ozone precursors. The concentrations are the smallest in Obninsk, its station subject to urban conditions, where ozone can be destroyed in emissions from plants and in vehicular exhausts. Both background (Vyatskiye Polyany and OPTEC-Karelia), and urban (RUDN) and suburban (Tropospheric Ozone Research (TOR)) stations become the members of the group with medium values of ozone concentrations. Stations in the group with minimal values line up in a similar order. Here, again, there are urban (OPTEC-N, -P, and -PR, Ulan-Ude, and Obninsk), suburban (Tarusa), and background (Fonovaya Observatory) stations.

It also follows from Fig. 1 that there is no longitudinal or latitudinal dependence of the annual averages, possibly due to the contribution from local sources of ozone precursors and anthropogenic factors. Also, there can be a contribution from long-term interannual variations in ozone concentration, when the annual average concentration can vary by as much as a factor of four [36]. A separate study is required to answer this question.

### 2.2 Annual Behavior of Ozone Concentration

**Fig. 2.**
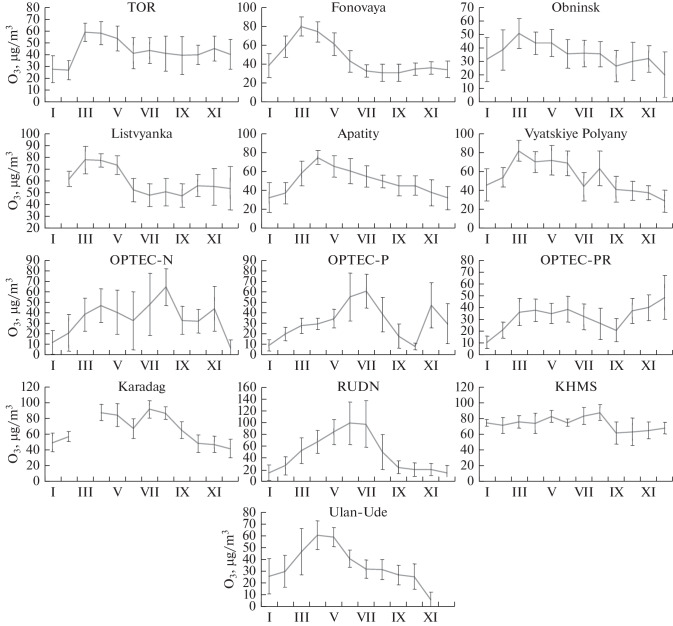
Annual behavior of ozone concentration at Russian stations based on monthly average data.

Monthly average data are used to consider the variations in ozone concentration at stations that operated throughout the year (Fig. 2).

From Fig. 2, it can be seen that the main concentration maximum in annual behavior is observed in spring at seven stations (TOR, Fonovaya, Listvyanka, Obninsk, Apatity, Vyatskiye Polyany, and Ulan-Ude), usually classified as background or suburban. The springtime concentration maximum was also recorded at a number of other stations [37–39]. Based on 20‑year monitoring, the springtime maximum in Tomsk was observed in 88.5% of cases [40]. Its specific feature is that it does not coincide in time with the maximum of incoming solar radiation. Considering that ozone is photochemically produced, this is not explainable yet [41]. Based on 11-year monitoring, the springtime maximum in Ulan-Ude was observed in 27.3% of cases [42].

Considering that organic gases may account for more than a half of the initial volume of ozone precursors in background regions [43, 44], a more probable process seems to be that associated with the springtime intensification of vegetation activity of plants delivering organic gases, i.e., ozone precursors [45, 46].

For stations located in St. Petersburg and Moscow (OPTEC-N, OPTEC-P, and RUDN), the main maximum of ozone concentration in 2021 was observed in July under the conditions of a blocking anticyclone and anomalously hot dry weather, associated with the large-scale circulation. The climatic annual maximum of surface ozone in midlatitudes and, in particular, in Tomsk and Moscow, is usually observed in spring (April–May). This was recently illustrated through the analysis of data from a continuous monitoring in 2005–2020 in [47]. In megalopolises in the southern latitudes, the ozone maximum occurs in July because of photochemical ozone production from anthropogenic emissions [48, 49].

There are two concentration maxima at the OPTEC-PR station. The first (not primary) maximum is recorded in the spring–summer period, and the second maximum is in December. This fact is difficult to explain. Possibly, it is associated with any local sources of ozone precursor gases, because no concentration increases were recorded in that period at the other St. Petersburg stations.

An extraordinary annual behavior is observed at KHMS (Fig. 2). The increased monthly average values are usually observed at KHMS in spring (March–May) and summer (July–August) and do not coincide in time with the maximum of the sunshine duration [50]. This regular effect has been observed and confirmed since the beginning of measurements of surface ozone at KHMS in 1989. The springtime local maximum is also manifested at other high-mountain stations such as Jungfraujoch (JFJ), where the stratosphere-troposphere exchange and mountain-valley circulation also influence the ozone regime, in addition to photochemical processes. The ozone concentration at KHMS is minimal in fall–winter. The absolute hourly average maximum in 2021 (140 μg/m^3^) was on July 19; and on August 13 and 18, the hourly averages reached 120 μg/m^3^. Under high-mountain conditions, that high ozone concentrations at KHMS can be associated with the stratospheric intrusions to the free troposphere, followed by mixing in the zone of orographic disturbances and, in particular, during foehn formation [51]. These events are generally short-term, lasting from one to few hours. The increased concentrations can also be associated with ozone production in polluted air during long-range transport.

**Fig. 3.**
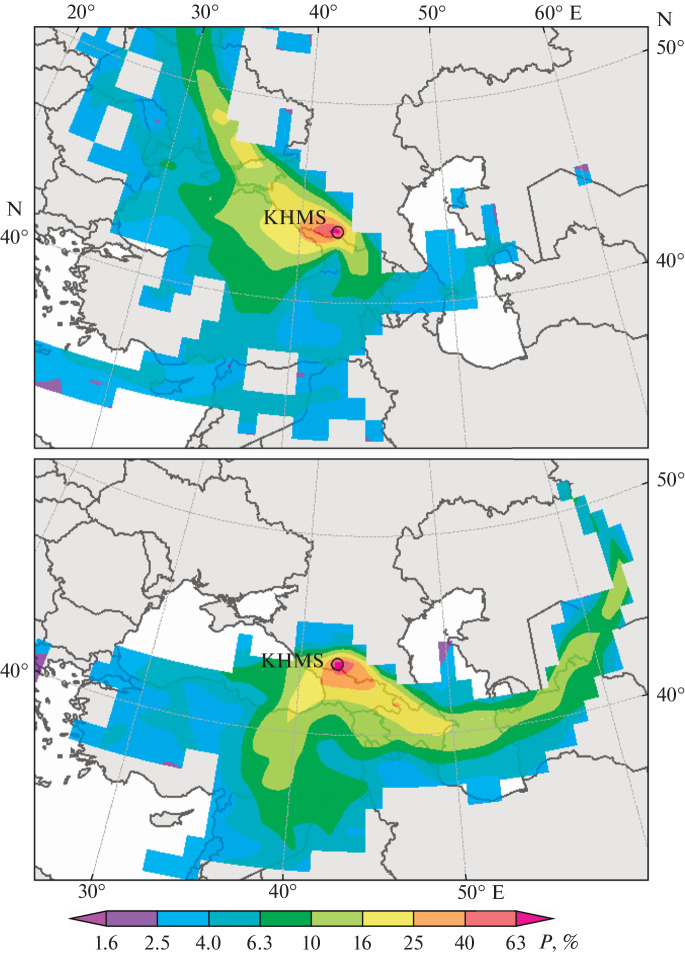
Transport probability of air particles (elementary air masses) associated with 10% of the lowest (upper panel) and 10% of the highest (lower panel) anomalies of surface ozone concentrations at KHMS in 2021 over different territories.

The trajectory analysis of air masses coming to KHMS was carried out to consider the contribution of long-range transport to the extreme values observed. The method for calculating the 7-day back trajectories was described in [30]. The 2021 measurements contain gaps, for technical reasons; therefore, fewer trajectories than in 2020 (∼17 000) were simulated. In contrast to urban conditions, where decreases in ozone concentration down to very low values signify strong pollution by nitrogen oxides, the anomalously low ozone concentrations at KHMS, which is located in a clean terrain and above the atmospheric boundary layer, are associated not so much with the long-range transport, but rather with dry deposition onto the Earth’s surface. This process is most active in a slowly moving air mass, where the contact of the analyzed air with the vegetation-covered surface is longest. The effect of pollutants transported from lower atmospheric levels from the direction of Kislovodsk (750–850 m ASL) on days with conditions favoring the development of the mountain-valley circulation, was shown in [50] to lead, on the contrary, to an increased daytime maximum, though not by a significant amount. Moreover, fogs favor the reduction of ozone concentration. Therefore, we excluded from analysis the back trajectories for days with a high (larger than 80%) humidity at the trajectory end point at KHMS. As a result, ∼13 000 trajectories remained in the dataset. This dataset was processed to select two sets of trajectories corresponding to extreme negative and extreme positive ozone anomalies, respectively, in the first and last deciles of the ozone anomaly distribution function, calculated with respect to the second-order polynomial fit. For extreme values of the ozone concentration anomalies of both signs, we retrieved the fields of the probability *P* (%) of air particle transport to KHMS in spatial grid cells 1° × 1° in size (Fig. 3).

On the whole, our results are consistent with the 2020 observations: extremely high anomalies of the surface ozone at KHMS in 2020 were associated with the southerly air transport and extremely low ozone was due to northwesterly transport. The extremely high anomalies in 2021 as compared to 2020 had an extra contribution from the southeastern transport direction: air particles associated with extremely high ozone anomalies in 2021 most probably moved not only over Turkey, as they did in 2020, but also over Azerbaijan, the Southern Caspian Sea, Turkmenistan, and Uzbekistan. Trajectories associated with extremely low surface concentrations in 2021, as in 2020, most probably passed over Krasnodar Krai, the Azov Sea, and the Ukrainian Azov region. That is, in the structure of the set of trajectories, we can distinctly single out two clusters: one associated with air masses coming from the Middle East, and the other (eastern) has a structure close to zonal. The trajectories of both types pass through the regions of intense oil and gas production and processing. If we consider the seasonal distribution of extreme ozone values, it is seen that the maximal concentrations are predominant in the spring–summer season, when there is a stable easterly transport associated with the Middle Asian anticyclone. Under the conditions of high temperatures and solar irradiance, the oxidation of volatile organic compounds in the plume from plants of the oil and gas industry leads to active ozone production and a stable increase in ozone concentration at KHMS.

### 2.3 Dynamics of Daily Average Concentrations

**Fig. 4.**
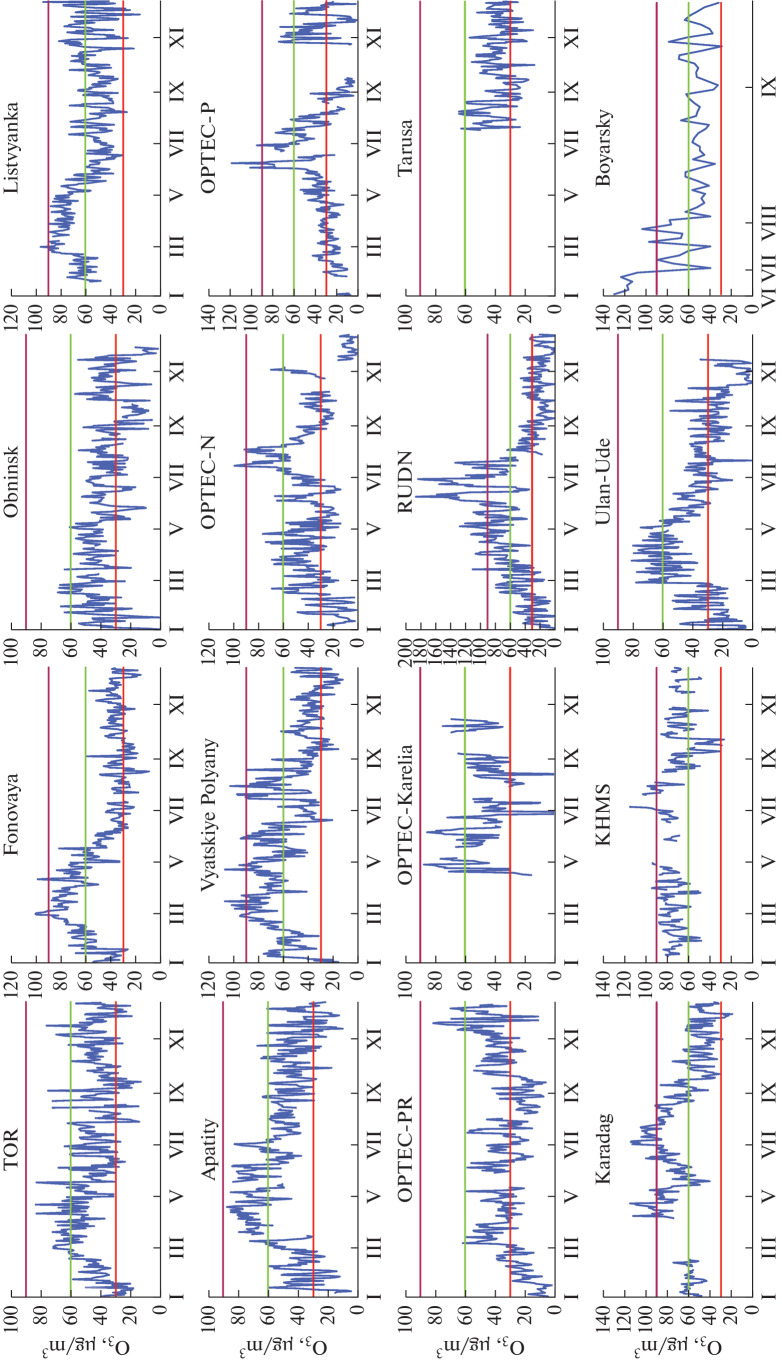
Daily average ozone concentrations at Russian stations in 2021; horizontal lines indicate 1MPC (red), 2MPC (green), and 3MPC (lilac).

One of the normalized characteristics of ozone content in air is the daily average ozone concentration, which should not exceed 30 μg/m^3^ [7]. The data on this characteristic are presented in Fig. 4.

It can be seen that the daily average MPC of ozone is exceeded at all observation sites for a major part of the year. We give below the numerical information on this. The MPC is exceeded two- and even three-fold at a number of stations. At urban stations, there are some days or short periods, usually during fall and winter, when the daily average ozone concentration decreases to zero. This seems to be due to low photochemical formation rate of ozone in these periods and its destruction by pollutants from automobile exhausts.

### 2.4 Daily Maximum Concentrations

**Fig. 5.**
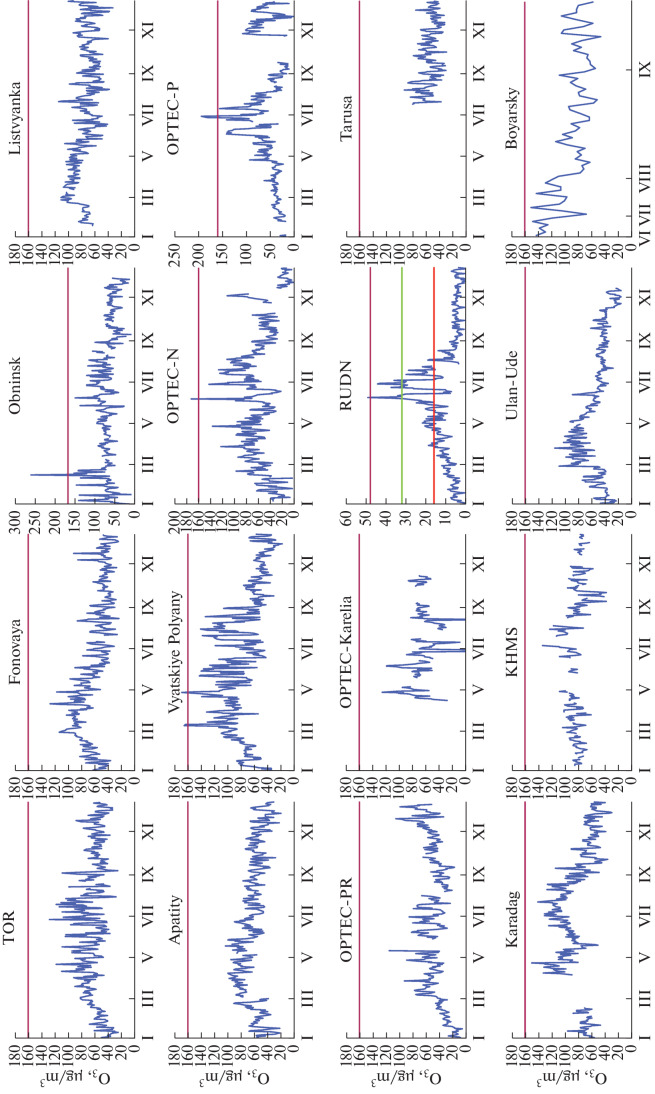
Maximal hourly average ozone concentrations; red horizontal lines indicate MPC_m.o_.

Still another normalized characteristic is the hourly maximal ozone concentration. Based on [7], it should not exceed 160 μg/m^3^. These data are summarized in Fig. 5.

From Fig. 5, it follows that the one-time maximum MPC was not exceeded at 9 out of 14 stations in 2021. Four stations recorded a onefold excess over MPC_m.o_. A threefold excess was recorded in Moscow at RUDN station, the pollution of which can be considered to be as strong as photochemical smog.

Thus, the regime of surface ozone in the Moscow region in 2021 differed from previous years in the following. First, in the concentrations largest on record and not observed before. Second, in the number of ozone episodes with characteristics exceeding Russian standards. Third, in the development of a summertime maximum that became the primary maximum in the annual behavior of surface ozone [52–54]. This markedly differs from data presented in previous reviews [29, 30]. In 2020, the weather in Moscow, which was rainier and colder than usual, was accompanied by decreased ozone concentrations, a poorly defined springtime maximum, and the absence of a summertime maximum. The specific features of large-scale circulation brought about a positive anomaly of air temperature in the warm period in April–August 2021 in the central regions of Russia. Data available at https://meteoinfo.ru/ indicate that the weather was 1–2° warmer than usual in spring and 3–4° warmer in summer. In summer months, the temperature of surface air rose above +30° on 31 days, 15 of which had temperatures as high as 33–36°. Hot dry weather favors intense photochemical ozone production [55–57]. It was the anomalous weather conditions that created prerequisites for the occurrence of high levels of surface ozone during summer.

The springtime maximum of surface ozone in Moscow was observed in April–May; the hourly concentrations of surface ozone at Mosecomonitoring AAPCS increased up to 130–150 μg/m^3^ on separate days. A prolonged springtime maximum in 2021 was determined by the specific features of its formation.

The summer ozone episodes have no analogs in the entire history of the regular ozone observations in Moscow [47]. In 2002 and 2010, in periods when smoke from forest fires influenced the ozone level, the high concentrations developed in late July–early August under the impact of long-range transport of ozone and its precursors from remote sources (see, e.g., [54]). In 2021, the main factor of anomalous growth of the surface ozone concentration had been an intense photochemical ozone production in air, polluted by local sources, under the conditions of atmospheric circulation weakened in blocking anticyclones.

**Fig. 6.**
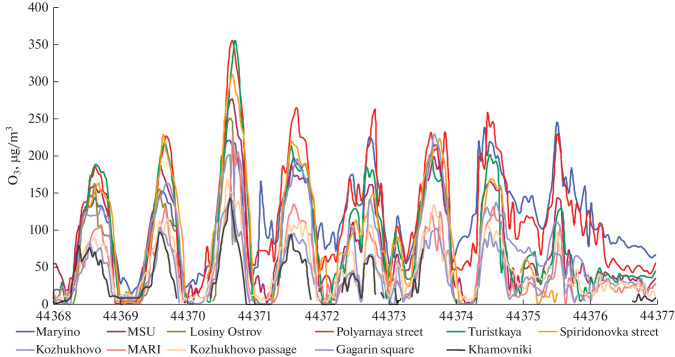
Surface ozone concentration at certain Mosecomonitoring stations in June 2021.

The longest ozone episode with extreme surface concentrations was observed in the second half of June under the conditions of the highest UV irradiance of the year. The two-week heat wave in June (since June 14, the air temperature rose above +25° at afternoon hours) was accompanied by severe radiation inversions and weak transport in the lower atmospheric layers; it reached its peak in the last week of the month. As shown in Fig. 6, the surface ozone concentration at certain AAPCSs exceeded the Russian standard (MPC_m.o_) by a factor of 1.2–1.6 for eight days; and on the days with extremely high pollution, the excesses were a factor of 1.8 (July 13) and a factor of 2.2 (June 23).

Using trajectory analysis, we found that ozone concentrations grew to extremely high levels in the air mass that circulated at the center of anticyclone over the Moscow agglomeration, thus favoring air loading by pollutants. The diurnal dynamics were maximal in this period. After the nighttime destruction/sink of ozone to 10–20 μg m^−3^, the concentrations rapidly grew up to 100–150 μg/m^−3^ and higher in the morning hours (from 08:00 to 12:00 LT). This process was accompanied by a rapid depletion of nitrogen oxides in air. At the same time, we note that the maximum increase in the surface ozone concentration in the June 23 episode coincided in time with the highest NO_2_ level characteristic of photochemical smogs [58–60].

**Fig. 7.**
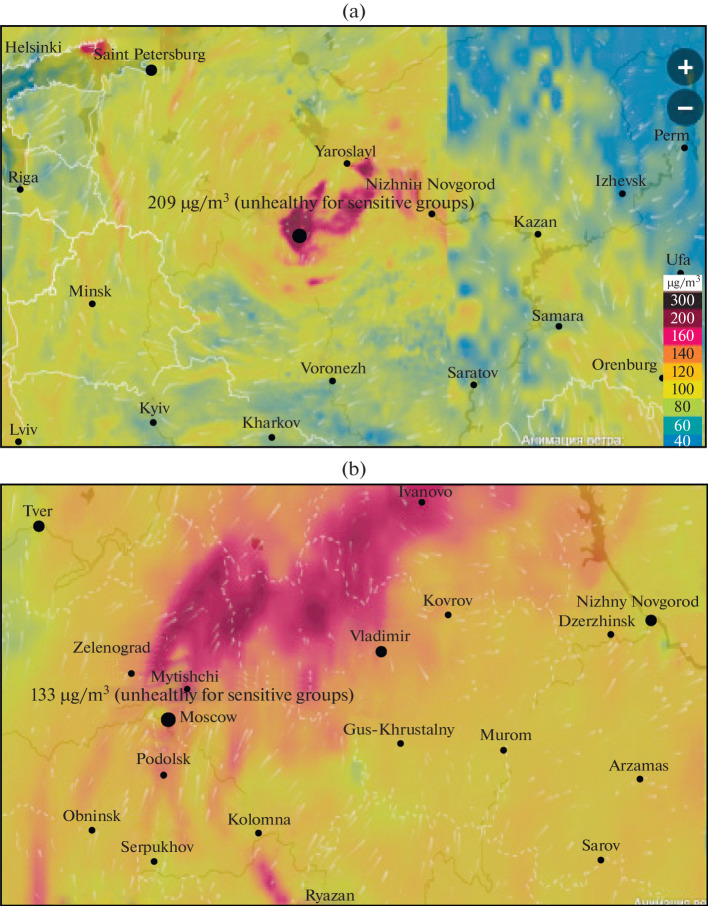
Surface ozone concentration on (a) June 23 and (b) 24, 2021, calculated from SILAM chemical transport model.

The plumes of polluted air with a high ozone content propagated long distances away from Moscow; calculations using SILAM chemical transport model (https://www.ventusky.com/) indicated that ozone-rich air masses moved to the neighboring regions; in particular, in the episode with the maximal ozone level in Moscow on June 23 and 24, the plume of anthropogenic ozone was carried northeast of Moscow, i.e., toward Ivanovo, Vladimir, and partly Nizhny Novgorod oblasts (Fig. 7).

In ozone episodes, the surface ozone field inside the megalopolis was characterized by high inhomogeneity: the difference in the maximal concentrations between urban and roadside stations reached 80–100 μg/m^3^ on certain days (Fig. 6). A prolonged ozone episode ended on June 28 after passage of a cold atmospheric front and change of air masses, as well as an inflow of clean air from the Baltic region.

The next ozone episode occurred in Moscow in July. It emerged against the background of a new wave of 30-degree heat on July 7–18 and was interrupted only on July 11 due to a short-term air inflow from the north. As in the June episode, the maximal increase in the level of ozone in the surface air was observed in the period from 15:00 to 17:00 LT, when air was maximally heated, the humidity was ∼30%, and the weather in the region was calm in the lower atmospheric layers with a slowly moving anticyclone above. As was shown in [61], low air humidity also favors an increase in ozone concentration. It is important that the NO_2_ concentrations at night maximally increased (to 100–120 μg/m^3^) precisely on July 8 and 13, thereby ensuring a high chemical activity in the morning hours for a daylight ozone buildup, and for ozone destruction in evening. We can also note that the level of surface ozone turned out to be ∼50 μg/m^3^ lower on July 13 at a temperature of +35° than it was on July 8 at a temperature of +32°, for all other atmospheric parameters remaining almost the same. The July ozone episode ended on July 19 owing to change of synoptic process and arrival of a clean air mass from the Baltic region.

In August, Moscow experienced another three short-term waves of 30-degree heat. However, no episodes of air pollution by ozone and its precursors occurred because of the absence of stagnant synoptic situations in those cases and a decrease in the level of UV radiation; only few urban stations recorded an atypical increase in ozone up to 0.8–0.9 MPC_m.o_.

The maximal surface ozone concentrations (SOCs) in 2021 at the (SBEM) Karadag background ecological monitoring station were observed on May 8 and August 6, on clear-sky wind-free days (150 and 141 μg/m^3^, respectively); and minimal SOCs, on December 17 (6 μg/m^3^), when the humidity was higher than 90%. In the summer period, the daily maximum ozone concentrations were observed under southerly and southeasterly winds, signifying the transport toward the AAPCS location from the direction of the sea. For the first time in the observation period since 2006, a morning ozone maximum was recorded on May 8. Three SOC peaks were noted on that day: the first at 04:00 LT, the second at 08:00 LT, and the third at 20:00 LT (131, 150, and 116 μg/m^3^, respectively). Presumably, the nighttime peaks are associated with the stratospheric ozone source, as well as with intense vertical mixing between the surface layer and the free troposphere.

Table 1 summarizes absolute maxima of ozone concentrations for each of the stations considered here.

**Table 1.** Absolute maxima of hourly averaged ozone concentrations in 2021 at Russian stations

Station	Concentration, μg/m^3^
Moscow, RUDN	490
Mosecomonitoring (northwest)	358
Obninsk	253
OPTEC-P	193
OPTEC-N	172
Vyatskiye Polyany	169
Boyarsky	151
Karadag	150
KHMS	134
TOR	129
Fonovaya	127
OPTEC-Karelia	126
OPTEC-PR	116
Ulan-Ude	116
Listvyanka	115
Apatity	104
Tarusa	92

## 3 OZONE DISTRIBUTION IN THE TROPOSPHERE

From July 1997 to the present, V.E. Zuev Institute of Atmospheric Optics, Siberian Branch, Russian Academy of Sciences (IAO SB RAS), has carried out monthly flights on the Optik aircraft laboratory to determine the vertical distributions of the gaseous and aerosol compositions of the atmosphere. The flights were first performed on an An-30 aircraft [62], and then on a Tu-134 aircraft [63]. The aircraft laboratory flies over the region of Karakan pine forest 100 km southwest of Novosibirsk to eliminate the urban contribution. The aircraft takes off at noon, when there is the maximal photochemical ozone production lasting for 2 h. The altitude range is from 0 to 7 km. Not all flights were performed in 2021 due to the coronavirus pandemic. Since, as discussed before [64, 65], ozone measurements under nonbackground conditions are problematic, three ozonometers are simultaneously operated onboard the aircraft: a chemiluminescent ozone analyzer 3.02P and two ultraviolet Thermo Environmental Instruments (TEI) model 49C UV ozone analyzers (United States). The ozonometers are pre-flight calibrated using ozone generator GS-2.

**Fig. 8.**
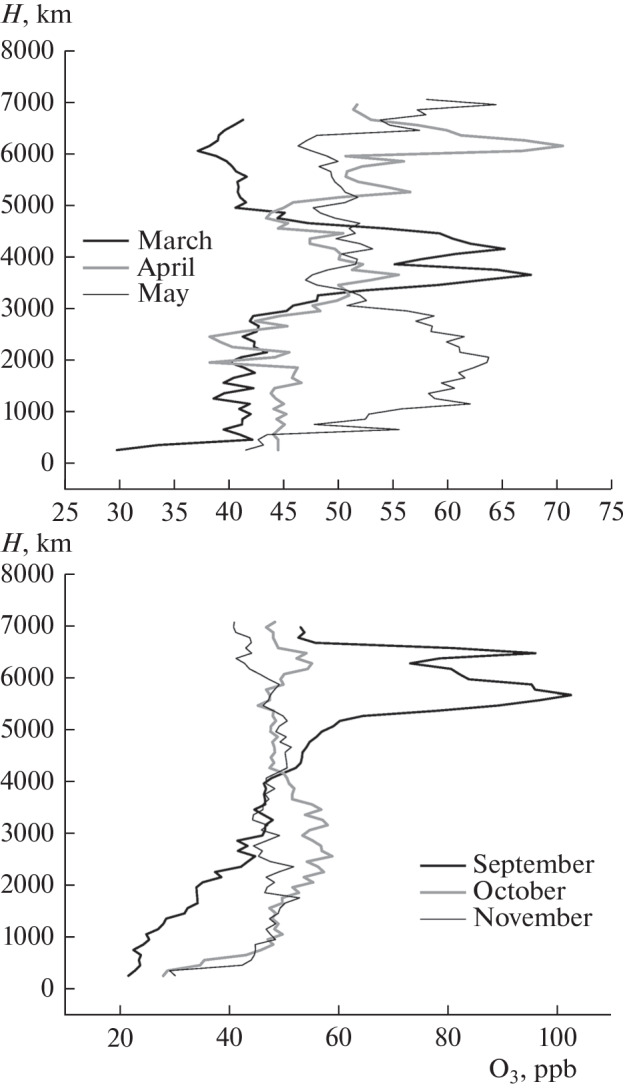
Vertical distribution of ozone concentration over Western Siberia in 2021.

The measurements of the vertical ozone distribution, shown in Fig. 8, show that no ozone is produced in the atmospheric boundary layer during the cold period (March), when the Earth’s surface is covered by snow. Ozone is produced only in May. Thus, О_3_ was mainly transported from the stratosphere. Note that the vertical distribution in the middle troposphere was near-neutral indicating that the ozone flux was not very intense.

The measurements in Fig. 8 strongly differ from long-term sounding results, which were summarized previously [66] for this same region. It was noted that there was almost constant photochemical ozone production in the surface or boundary layer of the atmosphere, which in 2021 was recorded as late as April.

**Fig. 9.**
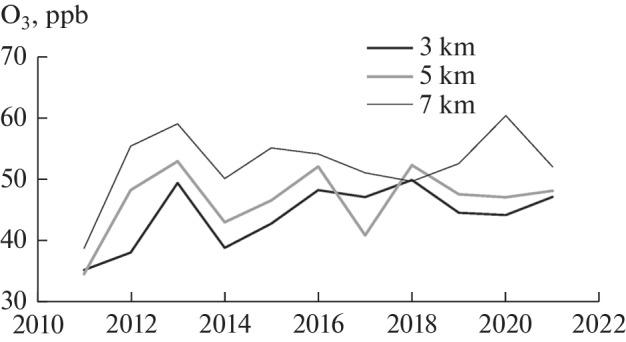
Variations in ozone concentrations at different altitudes over Western Siberia in 2011–2021.

Based on long-term measurements, Fig. 9 was plotted to identify and analyze the trends of variations in ozone concentration in the troposphere over Western Siberia noted in [34, 35]. This figure presents the annual average ozone concentrations at different altitudes.

From Fig. 9 it can be seen that the ozone concentration in the period of the coronavirus pandemic varies in opposite directions at the altitudes of the lower and upper troposphere. The variations are within the limits of long-term variability of the ozone concentration at these altitudes.

## 4 COMPLIANCE WITH HYGIENIC STANDARDS

The Russian Federation accepted the following standards regarding ozone concentration in the surface air layer [7]: 30 μg/m^3^ for the daily average maximum permissible concentration (MPC_d.a_), 160 μg/m^3^ for the one-time maximum permissible concentration (MPC_m.o_), and 100 μg/m^3^ for a duration of 8 h for the maximum permissible concentration of harmful substance in the air of a work zone (MPC_w.z_).

Table 2 summarizes the cases where the abovementioned MPCs were exceeded.

**Table 2.** Events of ozone concentrations above MPC in the surface air layer on the territory of Russia in the second half of 2021

Station	MPC_d.a_, 30 μg/m^3^			MPC_w.z_, 100 μg/m^3^	MPC_m.o_, 160 μg/m^3^
	1MPC, days/%	2MPC, days/%	3MPC, days/%		
OPTEC-PR	179/53.4	12/3.6	0/0	0	0
OPTEC-P	158/51.3	37/12.0	3/1.0	2	8
OPTEC-N	191/57.5	37/11.1	3/0.9	0	2
OPTEC-Karelia	117/90.0	30/23.1	0	1	0
SBEM Karadag	305/97.1	171/54.5	41/13.1	23	0
Obninsk	228/70.8	9/2.8	0/0	0	1
RUDN (Moscow)	212/58.9	115/31.9	55/15.3	145	402
KHMS	239/99.2	205/85.1	19/7.9	2	0
Vyatskiye Polyany	326/89.3	149/40.8	17/4.6	4	1
TOR station	306/84.8	51/14.1	0	0	0
Fonovaya	288/81.1	89/25.1	7/2.0	3	0
Listvyanka	327/98.2	157/48.0	2/0.6	0	0
Apatity	301/81.8	98/27.6	0	0	0
Tarusa	36/23.5	5/3.3	0	0	0
Ulan-Ude	191/57.2	36/10.8	0	0	0
Boyarsky	1/1.4	26/38.2	7/10.3	7	0

From Table 2 it can be seen that MPC_d.a_ could be exceeded in all regions where ozone was monitored. If KHMS is disregarded as a peculiar station, the frequency of occurrence of the daily average concentrations 30 μg/m^3^ and larger is within 23.5–97.1%. The concentrations 60 μg/m^3^ (2MPC) and larger also occur in all regions at a frequency ranging from 3.6 to 54.5%. Concentrations above MPC_w.z_ are recorded in a number of regions. The MPC_m.o_ is exceeded in five regions. It should be noted that two stations operated for a part of 2021; otherwise, the lower limits of the frequency of occurrence would be even higher. Of special note is the RUDN station, at which we recorded 145 periods longer than 8 h, when the concentration exceeded 100 μg/m^3^, and 402 cases with the hourly concentrations of 160 μg/m^3^ and larger. All features taken together, a classical photochemical smog persisted in Moscow for few days at afternoon hours. We note that the State Nature Organization Mosecomonitor regularly provides the information on ozone content in the surface air online at https://mosecom.mos.ru/vozdux/. However, residents know little about how hazardous ozone is for human health and what they should do during the events of hazardous ozone concentrations. More diseases, reported in numerous mass media, were merely attributed to high air temperatures in those periods of time.

## CONCLUSIONS

Our review shows that the ozone concentration in the surface air layer exceeded the national hygienic standards at all sites on the territory of Russia in 2021. This motivates the more comprehensive analysis of ozone precursors and development of measures for reducing their emission to the atmosphere.

It is also evident that the data in this review are mosaic in character. There are no data available for many big regions in the country. Therefore, there should be more cities and background regions where ozone would be regularly monitored.

The smog situation recorded during summer 2021 in Moscow can recur at any time under the conditions of the warming climate. This indicates that the system for warning the population about dangerous pollution of atmospheric air should be updated to include prognostic data on ozone concentration calculated using statistical and numerical models.
